# Genetic factors modify the effect of chronic viral infections on Alzheimer disease risk

**DOI:** 10.1002/alz.091644

**Published:** 2025-01-03

**Authors:** Marlene Tejeda, John J. Farrell, Congcong Zhu, Lee Wetzler, Kathryn L. Lunetta, William S. Bush, Eden R. Martin, Li‐San Wang, Gerard D. Schellenberg, Margaret A Pericak‐Vance, Jonathan L. Haines, Lindsay A. Farrer, Richard Sherva

**Affiliations:** ^1^ Boston University School of Medicine, Boston, MA USA; ^2^ Department of Medicine (Biomedical Genetics), Boston University Chobanian & Avedisian School of Medicine, Boston, MA USA; ^3^ Department of Biostatistics, Boston University School of Public Health, Boston, MA USA; ^4^ Cleveland Institute for Computational Biology, Department of Population and Quantitative Health Sciences, Case Western Reserve University, Cleveland, OH USA; ^5^ John P. Hussman Institute for Human Genomics, University of Miami Miller School of Medicine, Miami, FL USA; ^6^ Penn Neurodegeneration Genomics Center, Dept of Pathology and Laboratory Medicine, University of Pennsylvania, Philadelphia, PA USA; ^7^ Penn Neurodegeneration Genomics Center, Department of Pathology and Laboratory Medicine, University of Pennsylvania Perelman School of Medicine, Philadelphia, PA USA; ^8^ 1501 NW 10th Avenue, Miami, FL USA; ^9^ Case Western Reserve University School of Medicine, Department of Population & Quantitative Health Sciences, Cleveland Institute for Computational Biology, Cleveland, OH USA; ^10^ Boston University Alzheimer’s Disease Research Center, Boston University Chobanian & Avedisian School of Medicine, Boston, MA USA

## Abstract

**Background:**

Several viruses have been linked to Alzheimer disease (AD) by independent lines of evidence.

**Method:**

Whole genome and whole exome sequences (WGS/WES) derived from brain (3,404 AD cases, 894 controls) and blood (15,612 AD cases, 24,544 controls) obtained from European ancestry (EU), African American (AA), Mexican (HMX), South Asian Indian (IND), and Caribbean Hispanic (CH) participants of the Alzheimer’s Disease Sequencing Project (ADSP) and 276 AD cases 3,584 controls (all EU) from the Framingham Heart Study (FHS) that did not align to the human reference genome were aligned to viral reference genomes. A genome‐wide association study (GWAS) for viral DNA load was conducted using PLINK software and regression models with covariates for sex, age, ancestry principal components, and tissue source. A second GWAS tested association of the interaction of SNPs with total viral read count and specific viruses previously linked to AD (HSV‐1, HPV‐71, TTV‐10, and herpes and TTV clusters) with AD risk in five population ancestry groups. Results across subgroups (cohort, WES vs. WGS, tissue source, and ancestry) were combined by meta‐analysis using METAL software. P‐values for previously established AD risk SNPs were corrected for 83 tests and a standard genome‐wide significant (GWS) threshold of 5 × 10^‐8^ was used for GWAS analyses.

**Result:**

HSV‐1 was associated with an increased risk of AD in EUs (OR = 1.20, P = 3.23 × 10^‐6^) and AAs (OR = 1.18, P = 9.46 × 10^‐6^), but was protective in CHs (OR = 0.43, P = 5.06 × 10^‐62^). HPV‐71 was associated with a reduced risk of AD in EAs (OR = 0.94, P = 0.035). No significant virus‐AD associations were observed in individuals of HMX or IND ancestry. The interaction of a known AD risk SNP (rs6489896) in *TPCN1* with HSV‐1 was significantly associated with AD risk (OR_interaction = 1.11, P = 5.67 × 10^‐4^) in the trans‐ethnic meta‐analysis of all WGS samples. We identified GWS associations of 16 variants with viral reads in AAs. Several of these genes are involved in memory formation (KMT2A), synaptic transmission (*SLC28A2*), and immune response (*FCN2* and *CLEC12A*).

**Conclusion:**

These findings provide further evidence for the role of viral infections, particularly herpes viruses, in AD pathogenesis and highlight the importance of genetic factors in modulating these associations.

